# EGFR Activation Leads to Cell Death Independent of PI3K/AKT/mTOR in an AD293 Cell Line

**DOI:** 10.1371/journal.pone.0155230

**Published:** 2016-05-06

**Authors:** Cezary Treda, Marta Popeda, Magdalena Ksiazkiewicz, Dawid P. Grzela, Maciej P. Walczak, Mateusz Banaszczyk, Joanna Peciak, Ewelina Stoczynska-Fidelus, Piotr Rieske

**Affiliations:** 1 Department of Research and Development, Celther Polska Ltd., Lodz, Poland; 2 Department of Tumor Biology, Medical University of Lodz, Lodz, Poland; Hungarian Academy of Sciences, HUNGARY

## Abstract

The Epidermal Growth Factor Receptor (EGFR) and its mutations contribute in various ways to tumorigenesis and biology of human cancers. They are associated with tumor proliferation, progression, drug resistance and the process of apoptosis. There are also reports that overexpression and activation of wild-type EGFR may lead to cell apoptosis. To study this phenomenon, we overexpressed in an AD293 cell line two most frequently observed forms of the EGFR receptor: wild-type and the constitutively active mutant–EGFR variant III (EGFRvIII). Then, we compared the effect of EGF stimulation on cell viability and downstream EGFR signaling. AD293 cells overexpressing wild-type EGFR, despite a significant proliferation increase in serum supplemented medium, underwent apoptosis after EGF stimulation in serum free conditions. EGFRvIII expressing cells, however, were unaffected by either serum starvation or EGF treatment. The effect of EGF was completely neutralized by tyrosine kinase inhibitors (TKIs), indicating the specificity of this observation. Moreover, apoptosis was not prevented by inhibiting EGFR downstream proteins (PI3K, AKT and mTOR). Here we showed another EGFR function, dependent on environmental factors, which could be employed in therapy and drug design. We also proposed a new tool for EGFR inhibitor analysis.

## Introduction

Epidermal Growth Factor Receptor (EGFR) amplification or mutation occurs in many different tumors [1]. EGFR is a receptor tyrosine kinase (RTK) responsive to extracellular ligands such as EGF and TGF-α. One of its most frequent variants is Epidermal Growth Factor Receptor variant III (EGFRvIII), a truncated protein generated by in-frame deletion of exons 2–7, characterized by the absence of the ligand binding domain and constitutive, ligand-independent signaling [2]. Wild-type EGFR (EGFRwt) and EGFRvIII amplification frequently coincide [1,3], but whether this event influences a clinical outcome still remains unclear [4–6].

The biology of wild-type EGFR and EGFRvIII is complex. Both receptors have the same cytoplasmic domain and the differences in their oncogenic potential have been suggested to result from altered kinetics of signaling [7]. Also, not only activated EGFRwt and EGFRvIII differ in signaling patterns, but also EGFRwt shows constitutive activity that, in the absence of EGF, results in a third signaling pattern [8,9]. Finally, both receptors have been reported to interact with each other [10–12].

Various attempts have been made to overexpress EGFRwt and EGFRvIII, and study the activity of each variant separately or coexpressed [13–16]. The main obstacle in studying EGFRvIII is the fact that its amplicons are rapidly lost during culture [3]. Moreover, primary tumors or most cancer cell lines harbor many other mutations that obscure the actual activity of EGFRwt and EGFRvIII as well as their cooperation. Glioma cell lines U-87 MG and LN-229, most commonly used to study those receptors, harbor mutations of EGFR downstream pathways [17]. We decided to use AD293 cell line, a HEK-293 derivative with low mutation rate, improved adherence [18] and negligible endogenous expression of EGFR [19,20]. This cell line is a convenient expression tool for recombinant proteins due to its ability to perform most of the post-translational folding and processing as well as high cell division rate [21].

The selection of AD293 cell line as our experimental model, as well as the removal of interfering factors from the culture medium, would be particularly adequate for analysis of EGFR downstream pathway. Activation of EGFR leads to generation of phosphatidylinositol-3,4,5-trisphosphate (PIP3) [22], which in turn activates AKT, resulting in its phosphorylation at three regulatory sites: Thr308, Ser473 and Ser129 [23]. One of the major downstream effectors of AKT is the mammalian target of rapamycin (mTOR) complex 1 (mTORC1) [24]. The EGFR-PI3K-AKT-mTOR signaling cascade plays a central role in numerous cellular processes including metabolism, cell growth and proliferation, apoptosis, survival and differentiation, which contribute to tumor progression [25].

Albeit regarded as a mitogenic factor in most cell lines, EGF has been reported to induce apoptosis in EGFR-overexpressing cell lines: MDA-MB-468 and A431 [26–32]. In those cell lines, prolonged EGFR signaling and accumulation of receptor-ligand complexes creates a feedback mechanism, subsequently leading to induction of apoptosis. We observed that in AD293 cells cultured in serum free medium, EGF triggers apoptosis through overexpressed, stimulated EGFRwt. We compared the effect of EGFRwt stimulation and the action of constitutively active variant (EGFRvIII), and analyzed whether this process of programmed cell death may be reversed by known inhibitors of EGFR or inhibitors of its downstream signaling pathway, PI3K/AKT/mTOR.

## Materials and Methods

### Cell culture

AD293 cells were obtained from Agilent Technologies (United States). Cells were cultured in complete Dulbecco’s modified Eagle’s medium (Biowest, France) supplemented with 10% fetal bovine serum (FBS) (PAA, The Cell Culture Company, Austria), Penicillin-Streptomycin (Life Technologies, USA), Gentamycin (Biowest), Fungizone Antimycotic (Life Technologies), Primocin and Normocin (Invivogen, USA). Serum free conditions means a culturing in complete medium lacking FBS supplementation. Cultures were incubated at 37°C in humidified atmosphere and 5% CO_2_ and passaged with Trypsin/EDTA (0.05%; Life Technologies). Cells were observed with Biostation CT (Nikon, Japan) and cell counting was performed with ImageJ (http://imagej.nih.gov/ij/) and CL-Quant Ver3.10 imaging software (Nikon).

### Reagents and antibodies

Afatinib, erlotinib, gefitinib, GDC-0941, rapamycin and NU-7441 were purchased from Selleck Chemicals (USA). EGF (Invitrogen, USA) was used in concentration 20 ng/ml (unless other concentration is defined). The following primary antibodies were used: total EGFR antibody sc-03 (SantaCruz Biotechnology, USA), phospho-EGFR Y1173 (53A5), phospho-STAT5 Y694 (D47E7), phospho-AKT S473 (D9E) and AKT (#9272) (Cell Signaling Technology, USA), anti-Actin MAB1501 (clone C4) (Millipore, USA). Secondary antibodies—goat anti-rabbit IgG-HRP (sc-2004) and goat anti-mouse IgG-HRP (sc-2005), were purchased from SantaCruz. Annexin V-FITC Apoptosis Detection Kit, Hoechst 33342 and propidium iodide were purchased from Sigma-Aldrich (USA).

### Plasmid construction and transfection

The pIRESpuro plasmid (Clontech, USA) was digested with EcoRI/BamHI restriction enzymes (New England Biolabs, USA), blunt-ended with T4 DNA polymerase (NEB) and ligated with Gateway RfA reading frame cassette (Invitrogen). Afterwards, the CMV-GW-IRES-puro fragment was PCR-amplified (Primer sequences presented in Table 1), digested with HpaI/SnaBI restriction enzymes (NEB) and ligated with pLOC-RFP vector digested with Bsu36/SnaBI restriction enzymes (NEB). Final plasmid was named pLV1-puro-DEST. pIRES2neo3 (Clontech) was digested with EcoRI/NheI restriction enzymes (NEB), blunt-ended with T4 DNA polymerase (NEB) and ligated with Gateway RfA reading frame cassette (Invitrogen). Electrophoretic analysis and DNA sequencing were performed to confirm the recombinant vectors. Full length cDNA of EGFRwt and mutant EGFRvIII were amplified with primers containing attB1 and attB2 recombination sequences (Table 1), separated on agarose gel, purified with NucleoSpin® Gel and PCR Clean-up (Macherey-Nagel, Germany) and introduced to pENTR vector via BP reaction (Invitrogen). Next, the coding sequences were transferred to pLV1-puro-DEST under CMV promoter (EGFRwt) or pIRESneo3-DEST under CMV promoter (EGFRvIII) via LR reaction (Invitrogen). Both sequences were confirmed with Applied Biosystems 3130 Genetic Analyzer. The transfection was performed with Fugene HD (Promega, USA) with puromycin and neomycin (Invivogen) used to select cells, which successfully incorporated the plasmid. Each transfection resulted in generation of 2 separate clones (each derived from single cells), which were used for subsequent analyses.

**Table 1:** Primer sequences.

Primer	Sequence
pIRESpuro1-F	GACTTACGTACAGTTTGGTTAGTACCGGGCCGG
pIRESpuro1-R	GACTGTTAACGTCGGTGGGCCTCGGGGGCGGGTGCGGGG
EGFR-GW-F	GGGGACAAGTTTGTACAAAAAAGCAGCGTATGCGACCCTCCGGGACGGCC
EGFR-GW-R	GGGGACCACTTTGTACAAGAAAGCTGGGTTGCTCCAATAAATTCACTGC
HPRT1-F	GACCAGTCAACAGGGGACAT
HPRT1-R	AACACTTCGTGGGGTCCTTTTC
CyclinD1-F	CAATGACCCCGCACGATTTC
CyclinD1-R	CATGGAGGGCGGATTGGAA

### Western blot

Total cellular protein was isolated from cultures using RIPA Lysis and Extraction Buffer (Thermo Scientific, USA) supplemented with Halt Protease and Phosphatase Inhibitor Cocktail (Thermo Scientific), suspended in 4× Laemmli Sample Buffer (Bio-Rad Laboratories, USA) with β-mercaptoethanol (Sigma, USA) and boiled (98°C, 5 min). After separation in 8% SDS-polyacrylamide gel (Rotiphorese Ready-to-Use Gel Solutions; Carl Roth GmbH + Co. KG, Germany) the protein was transferred onto PVDF membrane (Bio-Rad Laboratories, USA) and then blocked with 5% PhosphoBlocker (Cell Biolabs Inc., USA). Opti-4CN Substrate Kit (Bio-Rad Laboratories) was used for HRP visualization. Visualized bands were analyzed with ImageJ and normalized to Actin bands.

### RNA isolation, reverse transcription and Real-time PCR

Total RNA was isolated using NucleoSpin RNA Kit (Macherey-Nagel) according to the manufacturer’s protocol. RNA concentrations were measured spectrophotometrically (NanoPhotometer Pearl, Implen GmbH, Germany). Total RNA was reverse transcribed using a QuantiTect Reverse Transcription Kit (Qiagen, Germany) according to the manufacturer’s protocol. Quantitative Real-Time PCR reactions were performed using SYBR® Select Master Mix (Life Technologies) in StepOnePlus Real-Time PCR System (Applied Biosystems, USA) to determine expression of *Cyclin D1* and *HPRT1* genes (Primer sequences presented in Table 1). *HPRT1* gene was used as a reference gene. The cycling conditions were as follows: 2 min at 50°C (UDG activation), 10 min at 95°C (polymerase activation) followed by 40 cycles of: 15 s at 95°C (denaturation), 30 s at 60°C (annealing) and 30 s at 72°C (extension). Real-time PCR efficiency and the relative expression were calculated using LinReg software and the method described by Pfaffl *et al*. [33], respectively.

### Apoptosis assay by flow cytometry

Cells were plated on 10-cm Petri dishes in complete medium. After 24 hours culture medium was replaced with serum free medium supplemented with DMSO, EGF (20 ng/ml), erlotinib (15 mM) or both EGF and erlotinib. 48 hours later floating cells were collected, the remaining cells were washed with PBS and harvested with Trypsin/EDTA (Life Technologies). Floating and adherent cells were pooled, washed with PBS, suspended in PBS (200 μl) and stained with Annexin V-FITC Apoptosis Detection Kit (Sigma Aldrich) according to manufacturer’s protocol. Cell suspensions were analyzed with FlowSight cytometer and visualized with IDEAS v6.1 software (Amnis Corporation, USA).

### Fluorescence microscopic assay for apoptosis

Cells were plated in complete medium. After 24 hours culture medium was replaced with serum free medium supplemented with DMSO, EGF (20 ng/ml), erlotinib (15 μM) or both EGF and erlotinib. The staining technique described herein was modified from Hoorens *et*. *al*. [34]. After 24 hours incubation cells were washed with PBS and stained with Hoechst 33342 (5 μg/ml; 3 minutes). Subsequently, staining solution was removed and propidium iodide (PI) in PBS was added (10 μg/ml; 3 minutes). Thereafter, second staining solution was removed and cells were immediately photographed by Eclipse CiS (Nikon) fitted with NIS Elements v4 (Nikon). Data were compiled from three different fields (40× magnification). Hoechst 33342 freely passes the plasma membrane, enters cells either with intact membranes or cells with damaged membranes and stains the condensed chromatin in apoptotic cells more brightly than the chromatin in normal cells (excitation/emission maxima 350/461 nm, blue when bound to DNA), whereas propidium iodide, a highly polar dye which is impermeable to cells with preserved membranes, stains DNA (excitation/emission maxima 535/617nm, red when bound to DNA). Viable or necrotic cells were identified by intact nuclei with, respectively, blue (Hoechst 33342) or yellow (Hoechst 33342 plus PI) fluorescence. Apoptotic cells were identified by fragmented nuclei, which exhibited either a blue (Hoechst 33342) or yellow (Hoechst 33342 plus PI) fluorescence depending on the stage in the process of apoptosis. In early apoptotic cells, only Hoechst 33342 reaches the nuclear material, while in the late apoptosis PI also penetrates the cells, generating a yellow signal [34].

### Statistical analyses

The studies were performed in triplicates and presented as the average values ± standard deviation. Difference between samples was compared by the two-tailed Student test and considered statistically significant at a P value of less than 0.05.

## Results

### Wild-type EGFR significantly increases cell proliferation

**Fig 1:**
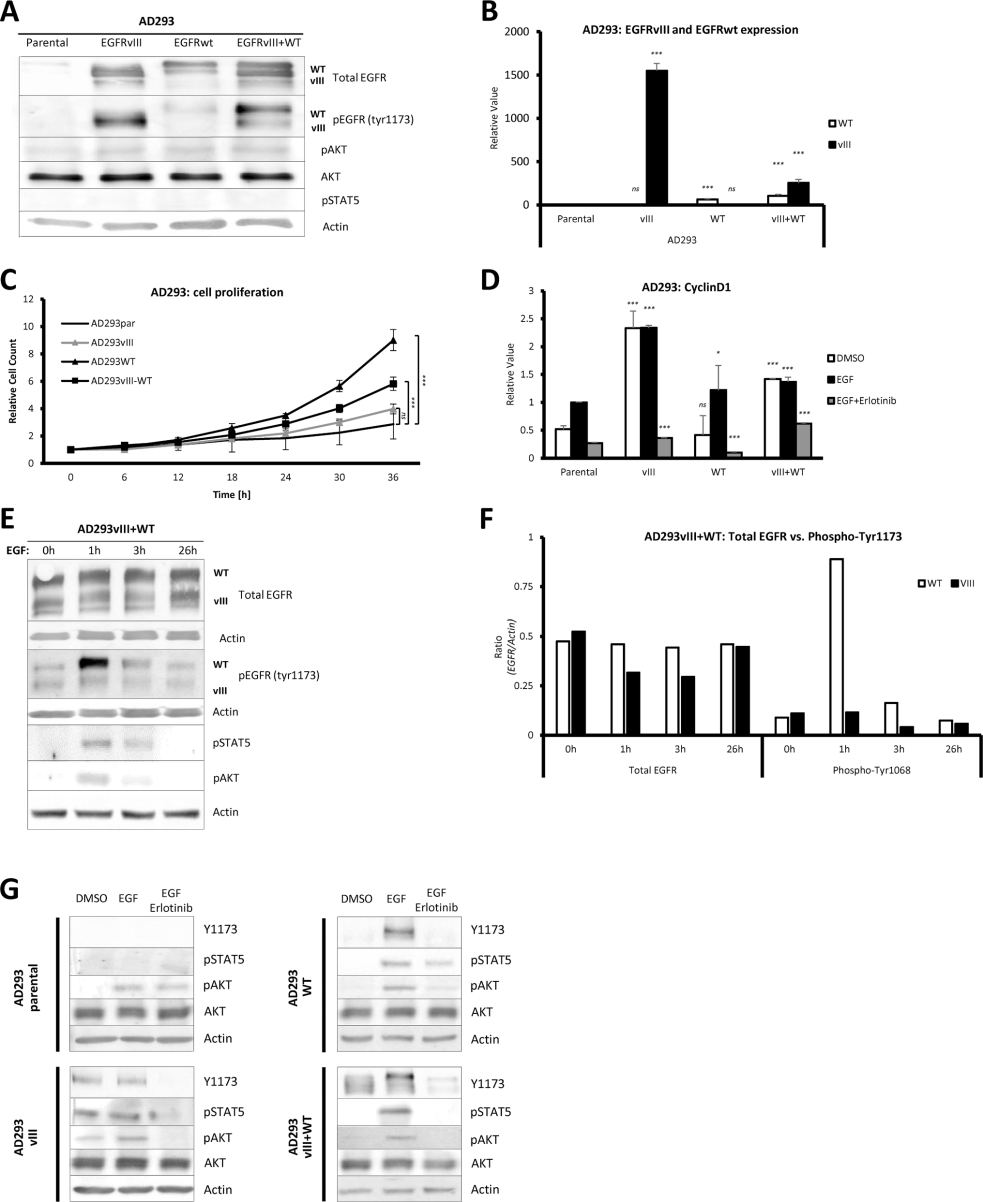
Wild-type EGFR increases proliferation rate in complete medium. (A,B) AD293 cells overexpress EGFRvIII, EGFRwt or both receptor variants. Cells were cultured in complete medium, collected and lysed. Western blots show levels of protein expression of total EGFR (sc-03 antibody), phospho-EGFR (tyr1173 antibody), phospho-AKT, AKT, phospho-STAT5 and Actin in four different cell lines (A). Real-time PCR was used to evaluate relative expression of total EGFR (wt and vIII) against *HPRT1* housekeeping gene in AD293 cell lines (B). *Statistical significance calculated against a value of specific gene in AD293par*, *ns*: *P>0*.05; ***: *P<0*.*001*. (C) EGFRwt expressing cells have the highest proliferation rate. Cells were seeded in complete medium and after 6 hours were moved to Nikon BioStation CT for live observation. Student’s t-test: ns: P>0.05; ***: P<0.001. (D) EGFRvIII expressing cells have constitutive expression of cyclin D1 independently on EGF treatment. Cells were serum starved for 24 hours, then supplemented with DMSO (control), EGF (50 ng/ml) or EGF/erlotinib (15 μM). After 24 hours cells were lysed and real-time RT-PCR was conducted. *Statistical significance calculated against a value for AD293par from the same group*, *ns*: *P>0*.05; *: *P<0*.05; ***: *P<0*.*001*. (E, F) EGFRwt has a peak activity after 1 hour of EGF treatment. Cells were serum starved for 24 hour and then serum free medium with EGF (20 ng/ml) was added. After specified time cells were lysed and blotted for total EGFR, phospho-EGFR and Actin (E). Bands intensity was calculated with ImageJ software (F). (G) AD293 cells overexpressing EGFRvIII have constitutively activated AKT and STAT5, while EGFRwt induces such activation after EGF treatment. Cells were serum starved for 24 hour and then serum free medium with EGF (20 ng/ml) or EGF/erlotinib (15 μM) was added. After 1 hour cells were lysed and blotted for phospho-EGFR, phospho-AKT, AKT, phospho-STAT5 and Actin.

AD293 cells, originally with negligible levels of EGFRwt (AD293par), were transfected with three plasmids to obtain three new cell lines: overexpressing EGFRwt, EGFRvIII and coexpressing both variants–EGFRwt and EGFRvIII (EGFRvIII+WT). Receptor expression in each cell line, cultured in complete medium, was confirmed by western blot and real-time PCR (Fig 1A and 1B). In complete medium, phosphorylation of EGFR at tyrosine 1173 was observed in all modified cell lines; importantly, EGFRwt phosphorylation was almost absent (Fig 1A). Western blot analysis revealed lack of STAT5 phosphorylation, while AKT phosphorylation was detectable but low in all tested cell lines (Fig 1A). MLPA analysis of cancer hotspots (p294 kit) revealed no alteration compared to parental cell line (S1A–S1D Fig).

In terms of proliferation in complete medium, we observed that EGFRwt-expressing cells had the highest division rate (P<0.001), substantially exceeding cells with EGFRvIII+WT (P<0.001), as well as similarly proliferating EGFRvIII and parental cells (Fig 1C). In serum free medium, parental and EGFRwt-expressing AD293 cells had increased expression of cyclin D1 after EGF stimulation (Fig 1D). On the other hand, cells with only EGFRvIII constitutively produced high levels of cyclin D1, independently of EGF treatment (Fig 1D). Importantly, a reversible tyrosine kinase inhibitor erlotinib significantly blocked cyclin D1 expression in all of the analyzed cell lines (P<0.001), indicating the dependence of cyclin D1 expression on EGFR signaling (Fig 1D).

### Wild-type EGFR phosphorylation peaks one hour after EGF stimulation

To analyze the dynamics of EGFR activation, we used AD293 cells with both receptor variants–EGFRwt and EGFRvIII. After 24-hour serum starvation we could still detect EGFRwt phosphorylation, which we regarded as a baseline (Fig 1E and 1F). We observed that EGFRwt phosphorylation was the strongest 1 hour after EGF stimulation, while in the 3rd hour it returned to the baseline indicating dephosphorylation without visible receptor degradation (Fig 1E and 1F). EGFRwt peak activity resulted in significant phosphorylation of STAT5 and AKT, and consistent with EGFR dephosphorylation, STAT5 and AKT bands also decreased (Fig 1E). Conversely, EGFRvIII was constitutively active during the whole assay, but it was found to have no influence on either STAT5 or AKT, most probably due to lower activation than EGFRwt (Fig 1E).

Recent reports have suggested that EGFRvIII promotes tumor growth and progression *via* constitutive activation of PI3K/Akt pathway [35,36]. Western blot analysis showed that AKT is active in EGF untreated EGFRvIII-expressing cells, but was not detected in unstimulated EGFRvIII+WT cells (Fig 1G). We also noticed that STAT5, like AKT, was active in AD293 cells overexpressing EGFRvIII without EGF treatment (Fig 1G), indicating EGFRvIII is able to activate STAT5 independent of EGFRwt. EGF treatment boosted EGFRwt, STAT5 and AKT phosphorylation in EGFRwt-overexpressing cell lines; however, EGF had no influence on only EGFRvIII-expressing cell lines (Fig 1E). Interestingly, STAT5 phosphorylation was undetectable in either stimulated or unstimulated AD293par (Fig 1G). Application of 15 μM of erlotinib significantly decreased phosphorylations of EGFR (both EGFRwt and EGFRvIII), AKT and STAT5 in all modified cell lines. The only exception was AD293par, in which EGFR phosphorylation was undetectable and the AKT activity was most probably not associated with EGFR signaling (Fig 1G). Finally, analysis of expression of other ErbB family RTKs that could influence EGFR dimerization and downstream signaling revealed insignificant endogenous expression of those receptors at both mRNA (S2A Fig) and protein level (S2B Fig).

### Wild-type EGFR activation induces cell apoptosis and loss of adhesion

**Fig 2:**
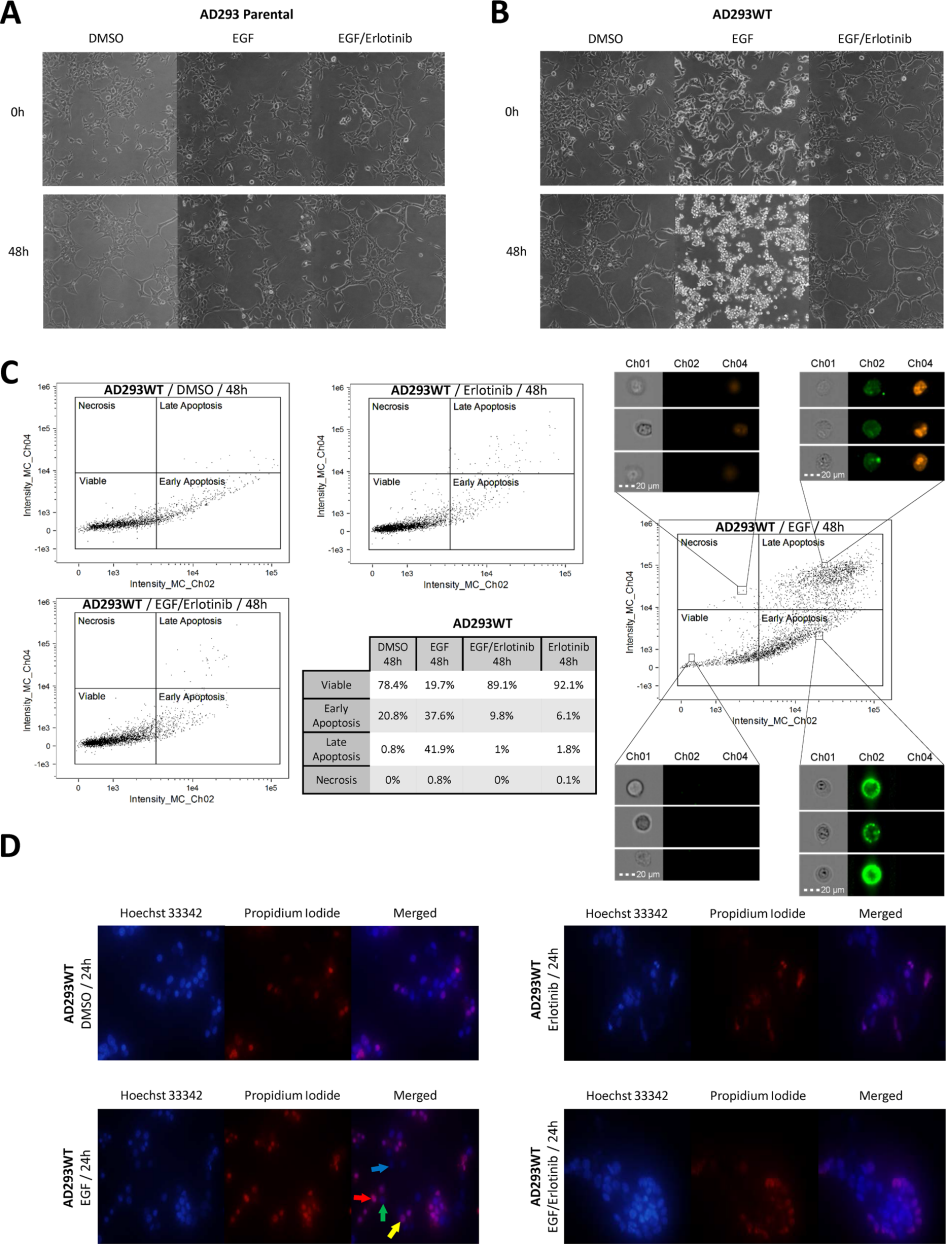
Serum starved EGF-treated cells expressing wild-type EGFR undergo apoptosis, what is prevented by erlotinib. (A, B) AD293par cells survive EGF treatment in contrast to massively detaching cells overexpressing EGFRwt. Medium was changed to serum free, DMSO/EGF/EGF+erlotinib was added and cells were photographed every 6 hours. Photos present cells at time 0 and at 48 hour. (C) Most of AD293 cells with wild-type EGFR treated with EGF become apoptotic. Medium was changed to serum free, DMSO/EGF/EGF+erlotinib/erlotinib was added, cells were harvested after 48 hours, stained with Annexin V FITC/propidium iodide and analyzed by flow cytometry. (D) Analysis of compacted state of chromatin in apoptotic cells confirmed the link between EGF activated EGFRwt and apoptosis. Medium was changed to serum free, DMSO/EGF/EGF+erlotinib/erlotinib was added, cells were stained after 24 hours with propidium iodide and Hoechst 33342 and analyzed under fluorescence microscopy. *Blue arrow*: *viable cell; green arrow*: *early apoptotic cell; yellow arrow*: *late apoptotic cell; red arrow*: *necrotic cell*.

We observed that cells overexpressing EGFRwt, placed in serum free medium, detach after stimulation with EGF, whereas stimulated parental cells remain intact (Fig 2A and 2B). To further analyze this phenomenon we stained EGFRwt cells with Annexin V and propidium iodide and examined them using an Amnis FlowSight cytometer (Fig 2C). We found that a small number of AD293wt cells become apoptotic, due to serum removal, but the addition of EGF caused massive apoptosis (21.6% of DMSO treated vs. 80.3% of EGF treated). Erlotinib prevented apoptosis regardless of EGF presence (Fig 2C).

Microscopic observation of cells stained with Hoechst 33342 and propidium iodide further confirmed the aforementioned results. Serum starved EGFRwt-expressing cells treated with DMSO, erlotinib or EGF/erlotinib had intact blue fluorescent nuclei suggesting lack of apoptosis. However, 24-hour treatment with EGF resulted in early and late apoptosis, as well as, in rare cases, necrosis (Fig 2D), since we observed yellow fluorescence and fragmentation of nuclei. In contrast to flow cytometry assays, we did not analyze 48-hour EGF treated cells, because EGF caused massive cell detachment prior to this time point. Both flow cytometry and Hoechst 33342/PI staining were also performed for AD293 expressing EGFRvIII+WT, giving results similar to cells overexpressing only EGFRwt (S3A and S3B Fig).

**Fig 3:**
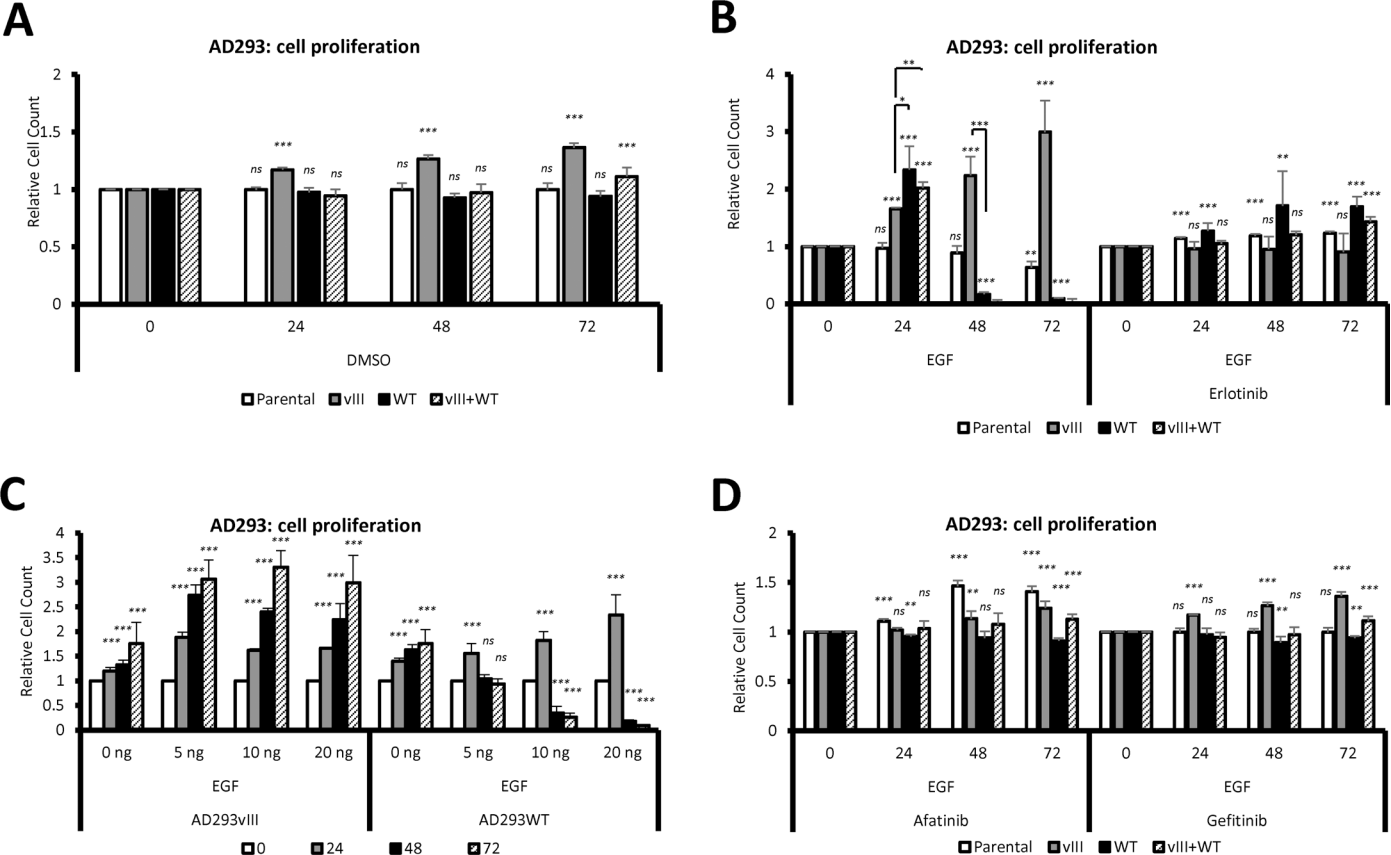
Tyrosine kinase inhibitors rescue EGF-treated cells expressing wild-type EGFR. (A) Unstimulated AD293 cells expressing EGFRvIII continue to proliferate in serum free medium. (B) AD293 cells expressing EGFRwt after addition of EGF proliferate fast during first 24 hours, then detach rapidly, what can be prevented by erlotinib. (C) An effective EGF concentration that induces cell detachment is 20 ng/ml. (D) Afatinib and gefitinib have similar effect to erlotinib. *Statistical significance calculated against time 0 within same cell line and same treatment*, *ns*: *P>0*.05; **: *P<0*.01; ***: *P<0*.*001*.

Our data indicated that, after serum withdrawal both parental and EGFRwt-expressing cells do not undergo cell death, rather they stop proliferating (Fig 2A and 2B; Fig 3A). However, cells expressing EGFRvIII and those with EGFRvIII+WT were still able to proliferate at a slow rate (Fig 3A). Moreover, serum-starved, EGF-treated EGFRvIII-expressing cells proliferated intensively (P<0.001), while EGFRwt and EGFRvIII+WT cells, despite an initial increase in number, were apoptotic after 48 hours and detached (Fig 2B and 2C; Fig 3B). EGF was ineffective when erlotinib, a reversible tyrosine kinase inhibitor, was also added. We observed that within 48-hour of EGF/erlotinib treatment EGFRwt-expressing cells increased in number and did not show cell detachment (Fig 2A and 2B; Fig 3B).

To determine the minimum concentration of EGF that would cause cell detachment, we compared AD293 expressing either EGFRvIII or EGFRwt. Addition of 5 ng/ml of EGF negatively influenced EGFRwt expressing cells, but the lowest concentration at which most of the cells detached was 20 ng/ml of EGF (Fig 3C). EGFRvIII expressing cells, similarly to previous attempts, were not influenced by EGF. We also checked other EGFR tyrosine kinase inhibitors, i.e. reversible tyrosine kinase inhibitor gefitinib and irreversible, covalent EGFR and erbB-2 (HER2) inhibitor–afatinib (Fig 3D). The effect of these two inhibitors was comparable to erlotinib and confirmed the link between cell apoptosis and EGFRwt activation. Furthermore, based on our observation that the phosphorylation of EGFRwt peaked 1 hour after EGF treatment and at the 3rd hour returned to the baseline (Fig 1E and 1F), we verified whether just one-hour stimulation was sufficient to determine the cell fate. We found that after 1 hour of EGF treatment EGFRwt-expressing cells initially behaved similarly to DMSO controls, but at the 48-hour time point, their number decreased (S4A Fig). On the other hand, EGFRvIII-overexpressing cells were not influenced by 1-hour EGF treatment (S4A Fig).

To conclude, EGF stimulation of cells overexpressing EGFRwt or both EGFRwt and EGFRvIII caused apoptosis and detachment. The negative influence of EGF was prevented by EGFR tyrosine kinase inhibition. Importantly, cells expressing EGFRvIII were not influenced by either serum starvation or EGF treatment.

### Analyses of the EGFR downstream signaling pathway did not reveal a culprit of EGF induced apoptosis

**Fig 4:**
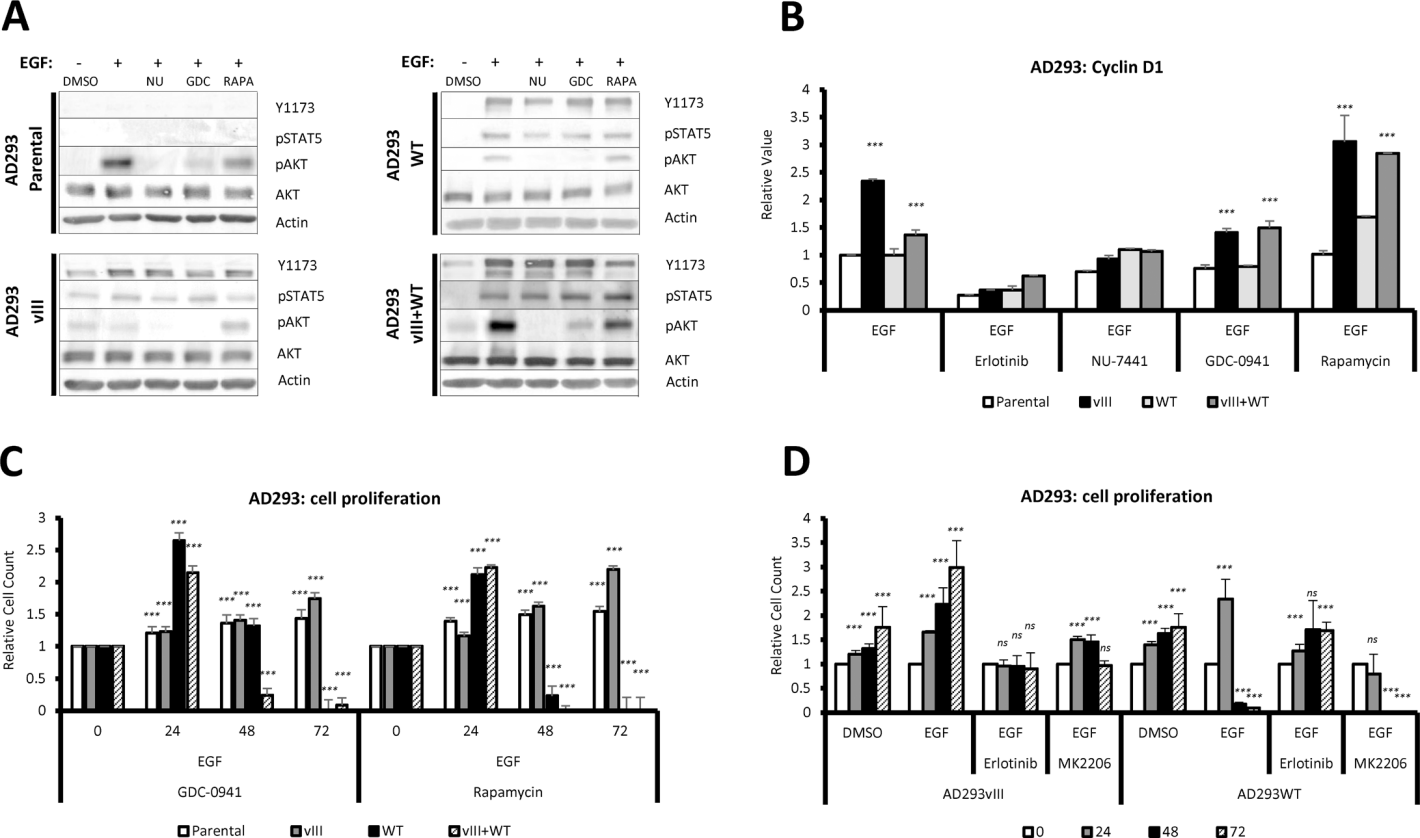
Inhibitors of PI3K, AKT and rapamycin do not prevent apoptosis in wild-type EGFR-expressing cells treated with EGF. (A) Rapamycin does not influence STAT5 and AKT activity, contrary to GDC-0941. Cells were serum starved for 24 hour and then serum free medium was supplemented with DMSO, EGF (20 ng/ml), EGF (20 ng/ml) + inhibitors (NU-7441 1 μM; GDC-0941 1 μM; rapamycin 1 μM). After 1 hour, cells were lysed and blotted for phospho-EGFR, phospho-STAT5, phospho-AKT, AKT and Actin. (B) PI3K inhibition slightly decreased and mTOR inhibition increased expression of cyclin D1. Cells were serum starved for 24 hours and DMSO, EGF (20 ng/ml), EGF (20 ng/ml) + inhibitors (erlotinib 15 μM; NU-7441 1 μM; GDC-0941 1 μM; rapamycin 1 μM) were added. After 24 hours cells were lysed and real-time RT-PCR was conducted. (C) PI3K and mTOR inhibitors do not reverse an effect of EGF. Medium was changed to serum free with DMSO/GDC-0941/rapamycin, and every 24 hours cells were photographed and counted. (D) AKT inhibitor MK2206 did not rescue cells after EGF treatment. Medium was changed to serum free and supplemented with EGF (20 ng/ml) and DMSO or inhibitors (erlotinib 15 μM or MK2206 2 μM). Cells, run in triplicates, were photographed every 6 hours and counted (time points shown: 0 h, 24 h, 48 h and 72 h). *Significance calculated against time 0 within same cell line and same treatment*, *ns*: *P>0*.05; ***: *P<0*.*001*.

We showed that EGFRwt triggers cell apoptosis upon ligand binding and subsequent activation of its tyrosine kinase domain. Therefore, we hypothesized that apoptosis might be alleviated by blocking EGFR downstream targets: PI3K (by GDC-0941), AKT (by MK2206), DNA-PK (by NU-7441) or mTOR (by rapamycin) (Table 2). We observed that inhibition of these proteins did not influence STAT5 phosphorylation (Fig 4A), suggesting possible direct interaction between STAT5 and EGFR. DNA-PK and PI3K inhibitors significantly decreased AKT phosphorylation, and rapamycin had no effect on the phosphorylation of AKT (Fig 4A). Further analysis of PI3K and mTOR inhibitors showed no significant influence on cyclin D1 expression (Fig 4B). Moreover, based on the reported physical interaction of EGFR with DNA-dependent kinase (DNA-PK) [37], we included the NU-7441 inhibitor in our experiment. However, supplementation of serum free medium with EGF and NU-7441 not only decreased cyclin D1 expression (Fig 4B), but also led to cell detachment within 24 hours, almost twice as early as after EGF treatment (S4B Fig).

**Table 2:** Effect of different inhibitors on serum starved, EGF treated, EGFRwt expressing AD293 cells.

Inhibitor name	Target	Effect on:			
		EGF treated EGFRwt cells	Cyclin D1 expression	AKT phosphorylation	STAT5 phosphorylation
Erlotinib	EGFR	Rescued	Decreased	Decreased	None
Gefitinib	EGFR	Rescued	ND^*^	ND^*^	ND^*^
Afatinib	EGFR/HER2	Rescued	ND^*^	ND^*^	ND^*^
GDC-0941	PI3Kα/δ	None	None	Decreased	None
Rapamycin	mTOR	None	None	None	None
NU-7441	DNA-PK	Deteriorated	Decreased	Decreased	None

* ND–not determined

Analyses of cell proliferation indicated that neither GDC-0941 nor rapamycin rescue cells exposed to EGF (Fig 4C). Moreover, initial proliferation observed within the first 24 hours in 1-hour EGF treated cells expressing EGFRwt was suppressed by PI3K inhibitors (S4A Fig). We also noticed that rapamycin protected EGFRwt-expressing cells from any negative influence of 1-hour EGF treatment, indicating a limited effect of this inhibitor (S4A Fig). Finally, the AKT specific inhibitor, which was ineffective in complete medium (S4C Fig), significantly impaired proliferation of cells in serum free medium (Fig 4D).

## Discussion

EGFR amplification and EGFRvIII rearrangement are the main oncogenic events in various tumors. In this study, we created three different cell lines to analyze EGFRwt and EGFRvIII, expressed separately or combined. We have demonstrated that those two receptor variants trigger different responses in AD293 cell line, frequently used as low tumorigenic for testing oncogenic properties of cancer-associated genes. We also found that EGFRwt is not critical for EGFRvIII activation, which supported findings by Ymer *et al*. [38] and contradicted those of Fan *et al*. [16].

EGFR and its related family members are present in various cancers and are associated with a poor prognosis for patient survival [39]. Indeed, we showed that EGFRwt overexpression significantly improves cell proliferation in complete medium, accompanied by a low activity of AKT and undetectable phosphorylation of STAT5. On the other hand, removal of serum and EGF treatment led to different signaling resulting in a massive cell detachment and apoptosis, accompanied by visible AKT and STAT5 activity.

Our observations confirm previous report on MDA-MB-468, a breast cancer cell line with elevated EGFR expression and mutation in *PTEN* gene [40]. In MDA-MB-468 cells, increased level of EGFR was not sufficient to induce apoptosis, but continuous treatment with EGF might have led to stronger receptor internalization and possibly intracellular accumulation of active receptors, causing cell apoptosis [26]. We analyzed this phenomenon in AD293 cell line and confirmed that 1-hour treatment with EGF, despite resulting in EGFR phosphorylation peak, did not generate a signal sufficient to induce cell death. However, prolonged treatment of EGFRwt with its ligand triggered cell detachment, indicating that apoptosis is not only linked with this receptor phosphorylation status, but also with the duration of stimulation. The aforementioned findings supported previous reports on gene expression patterns in EGFRwt whether unstimulated or time-dependent EGF stimulated [8,9]. Furthermore, two recently published reports by *Alanazi et al*. present microarray, proteomic and miRNA time course expression profiling of EGF treated A431 cell line, in which apoptosis was triggered as well [31,32]. It was found that EGF induces various anti- and pro-apoptotic signals, thus a dysregulation of different signaling pathways is responsible for the cell fate [31]. Alanazi *et al*. also showed a link between EGF treatment, cell detachment and apoptosis on mRNA and miRNA level [31,32], what is consistent with our results and would be an interesting subject of further investigation.

EGF treatment results in phosphorylation of EGFR. Activated receptors are internalized, then associate with numerous phosphorylated proteins and finally lose both phosphotyrosine and associated ligands just before degradation [41]. Burke *et al*. [41] also indicated that an activated receptor in the plasma membrane and internalized receptors had different signaling patterns, which is in agreement with our results. Furthermore, Nishimura *et al*. reported a reduction of EGFR internalization rate and a retarded transition from early to late endosomes/lysosomes upon gefitinib treatment [42]. This may support our observation on erlotinib, gefitinib and afatinib mediated prevention of detachment in ligand-stimulated EGFRwt-overexpressing cells.

EGF does not interact with EGFRvIII and in our study EGFRvIII-expressing cells treated with EGF continued to proliferate without any interruption. However, we noticed an improvement in the proliferation rate of EGF-treated EGFRvIII–expressing cells when compared to those treated with DMSO, which may suggest a marginal, but important EGF-related activity of the receptor in AD293 cells.

Recent findings indicate that cells derived from HEK293, HEK293-T Phoenix, which are widely used as retrovirus packaging cells, undergo massive cell death within 72 h after serum withdrawal [43]. This has been linked to an excess of nutrients, i.e. massive cell death in serum-free medium may be prevented by either glucose or amino acid withdrawal [43]. In the absence of serum, cells seem to be self-sufficient for mitogenic stimulation and robustly proliferate. In this regard, cell proliferation and death are simultaneous and mechanistically linked with each other [43,44]. We did not observe any adverse effects (cell detachment) of serum free medium on AD293 cell cultures. This could be due to the high level of expression of cell adhesion-related proteins [18]. Furthermore, activated receptor degradation has already been reported [45,46]; however, this was not visible in our study. We infer that it is the cause of either robust overexpression of the receptor or inefficient degradation of an internalized receptor in AD293 cells.

Consistent with previous observations [47], we noticed that STAT5, inactive in AD293par cells, was phosphorylated in EGFRvIII or ligand activated wild-type EGFR expressing cells. After the addition of erlotinib, STAT5 phosphorylation significantly decreased. Hung *et al*. reported that EGFR interacts with STAT5 on the ATRS motif to transactivate Aurora-A promoter, resulting in expression of Aurora-A encoding genes. This, in turn, results in the induction of centrosome amplification and microtubule disorder that may contribute to worse clinical outcomes [15]. However, our results indicated no connection between STAT5 phosphorylation and cell apoptosis, because phosphorylation patterns in either ligand activated EGFRwt or EGFRvIII were the same. STAT5 was also shown to be active independent of PI3K/AKT/mTOR, which indicated a direct correlation between STAT5 and EGFR, reported also elsewhere [16,48]. In this regard, we considered STAT5 phosphorylation as an additional control of a correct inhibition of EGFR tyrosine kinase with erlotinib.

Heimberger *et al*. suggested that ligand-activated EGFR translocates to the nucleus and associates with an A/T-rich sequence (ATRS) in the cyclin D1 promoter. This results in transcriptional activation of cyclin D1, which is required for cell cycle G1/S transition [6]. Indeed, we found that EGF treated wild-type EGFR caused increased cyclin D1 expression, but also EGFRvIII constitutive activity resulted in similar levels of cyclin D1 expression. In this regard, we did not observe any cyclin D1 influence on cell proliferation rates, despite the correlation between EGFR activity and cyclin D1 expression confirmed by erlotinib treatment.

Finally, activation of the PI3K/Akt/mTOR pathway results in a profound disturbance of the control of cell growth and survival, metastasis, angiogenesis, and therapy resistance, as summarized by Porta *et al*. [49]. Panieri *et al*. showed that mTOR inhibition protected cells from nutrient-induced cell death, and mTOR silencing also increased AKT phosphorylation; yet, it did not protect HEK293-T Phoenix cells from apoptosis [43]. However, we did not observe any influence of the inhibition of PI3K, AKT or mTOR signaling pathways on the apoptosis of EGFRwt expressing cells treated with EGF. Based on these findings, we infer that EGFRwt related cell death is neither nutrient-induced nor related to mitogenic signaling. In this regard, further experiments are necessary to determine if there is any effect of prolonged EGF stimulation of wild-type EGFR on receptor internalization and further signaling processes.

## Conclusions

1. AD293 cells, expressing wild-type EGFR and cultured in complete medium (supplemented with FBS), showed significantly increased proliferation rates as compared to parental or EGFRvIII-expressing cells.
2. Upon a lack of external stimuli (EGF), AD293 cells expressing EGFRvIII had active downstream signaling, AKT and cyclin D1, as well as showed a mitogenic signal. On the other hand, in complete medium these cells behaved similarly to the parental ones.
3. Serum starved, EGFRwt-overexpressing cells underwent cell death upon stimulation with EGF. This effect was mediated by EGFRwt kinase domain, but independently from the PI3K/AKT/mTOR axis. On the other hand, serum starved EGFRvIII-expressing cells maintained constant proliferation. These observations indicated a difference between the wild-type and mutant form of active receptors in downstream signaling.
4. AD293 cell line is characterized by a number of advantages, which make it an adequate model for research on novel EGFR inhibitors, as well as studies of EGFRwt/EGFRvIII signaling pathways. These include not only the possibility to overexpress functional proteins–EGFRwt or EGFRvIII, but also the high rate of cell division and ease of maintenance.

## Supporting Information

S1 Fig Parental AD293 cells and those with overexpressed EGFRwt and EGFRvIII have identical Gain/Loss profile. (A) AD293 parental cells. (B) AD293 with overexpressed EGFRvIII. (C) AD293 with overexpressed EGFRwt. (D) AD293 with overexpressed both EGFRwt and EGFRvIII.
(TIF)

S2 Fig Analysis of ErbB family RTKs. (A) ErbB family RTKs are expressed at a very low level in AD293 cell lines. Real-time PCR was used to evaluate relative gene copy number variation of total EGFR (wt, HER1), HER2, HER3 and HER4 against *HPRT1* housekeeping gene in AD293 cell lines. (B) Western blot analysis confirmed insignificant level of ErbB-3 (HER3) as well as lack of active/phosphorylated form of HER3. AD293 cells expressing EGFRvIII or EGFRwt were serum starved for 24 hours and DMSO/EGF/EGF+(Afatinib 500 nM)/Gefitinib 5 μM) were added. After 1 hour cells were lysed and blotted for phospho-EGFR, total EGFR, HER3, phospho-HER3, AKT and Actin. *Statistical significance calculated against values for any of 4 genes in AD293par*, ***: *P<0*.*001*.
(TIF)

S3 Fig Cells expressing EGFRvIII and EGFRwt undergo apoptosis after EGF treatment. (A) Most of AD293 cells expressing both EGFRvIII and wild-type EGFR become apoptotic when treated with EGF. Medium was changed to serum free, DMSO/EGF/EGF+erlotinib was added, cells were harvested after 48 hours, stained with Annexin V FITC/propidium iodide and analyzed by flow cytometry. As a control, untreated cells grown in complete serum (10% FBS added) were analyzed. (B) Analysis of compacted state of chromatin in apoptotic cells confirmed the link between EGF activated EGFRwt and apoptosis. Medium was changed to serum free, supplemented with DMSO/EGF/EGF+erlotinib/erlotinib, after 24 hour incubation cells were stained with propidium iodide and Hoechst 33342, and analyzed under fluorescence microscopy.
(TIF)

S4 Fig AKT inhibitor did not reverse the pro-apoptotic effect of EGF. (A) Despite the peak of EGFRwt activity was observed after 1 hour treatment with EGF, this event was not sufficient to induce apoptosis/detachment of the cells. Medium was changed to serum free and supplemented with EGF (20 ng/ml) and DMSO or inhibitors (GDC-0941 1 μM; rapamycin 1 μM). After 1 hour medium was changed again and all of the abovementioned supplements were used, with the exception of EGF. Cells were photographed every 6 hours and counted (time points shown: 0 h, 24 h, 48 h and 72 h). Done in triplicates. (B) NU-7441, DNA-PK inhibitor, induces robust cell detachment within 24 hours. Medium was changed to serum free and supplemented with EGF (20 ng/ml) and inhibitor NU-7441 (1 μM). Cells were photographed at 0 h and 24 h time points, each time at least 3 wells were evaluated. (C) MK2206, AKT inhibitor, has no significant influence on cell proliferation in complete growth medium. Cells were seeded onto 6-well plate in complete medium and after 6 hours were moved to Nikon BioStation CT for live observation.
(TIF)

S1 File Supplementary Materials and Methods. (PDF)

S1 Table Primer sequences. (PDF)
