# Supplementary figures and images for: EGFR Activation Leads to Cell Death Independent of PI3K/AKT/mTOR in an AD293 Cell Line
Source: PLoS One. 2016 May 6;11(5):e0155230. doi: 10.1371/journal.pone.0155230 (PMC4859505; doi:10.1371/journal.pone.0155230)

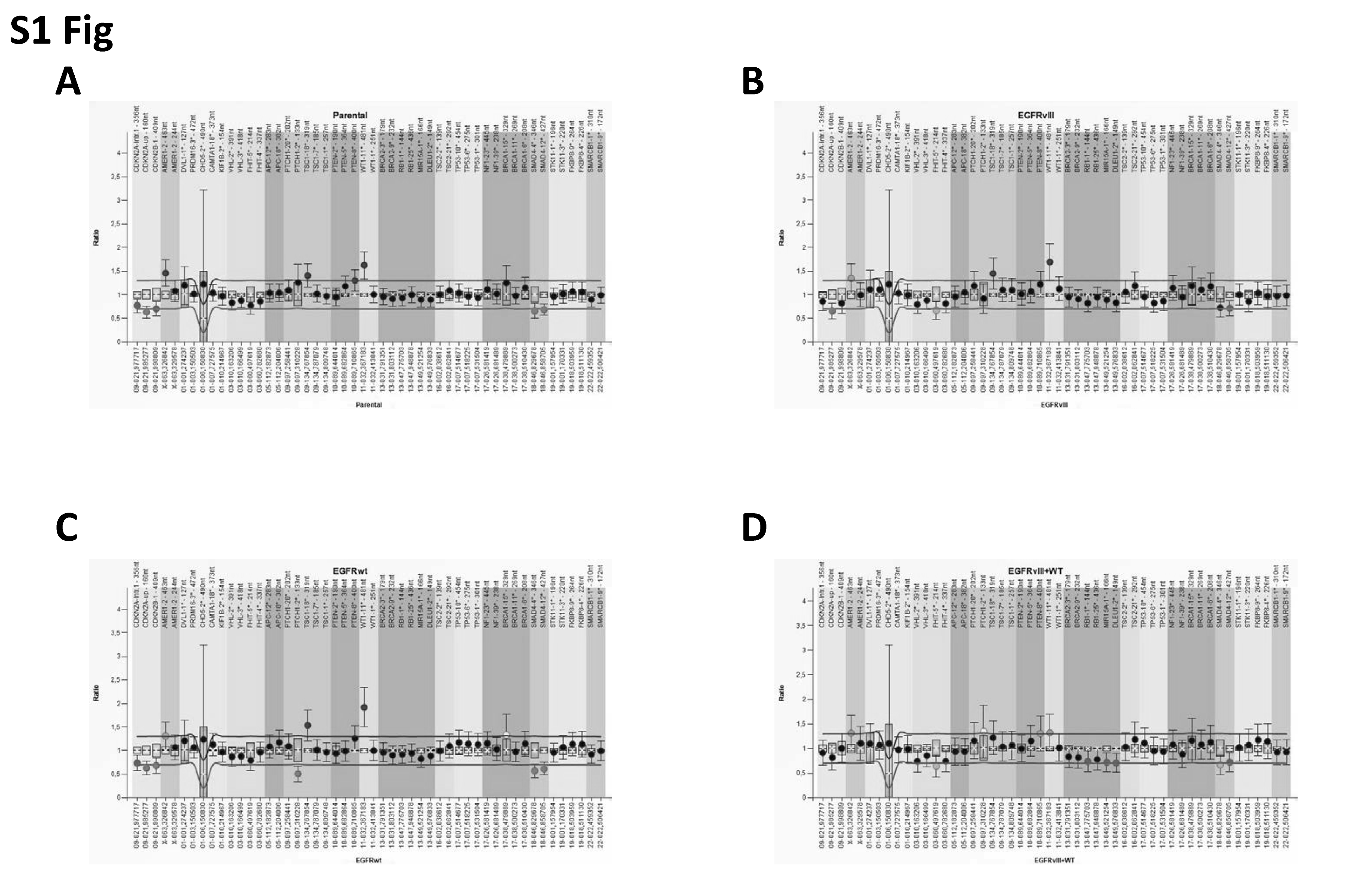

Supplement: S1 Fig — (A) AD293 parental cells. (B) AD293 with overexpressed EGFRvIII. (C) AD293 with overexpressed EGFRwt. (D) AD293 with overexpressed both EGFRwt and EGFRvIII. (TIF) [file pone.0155230.s001.tif]

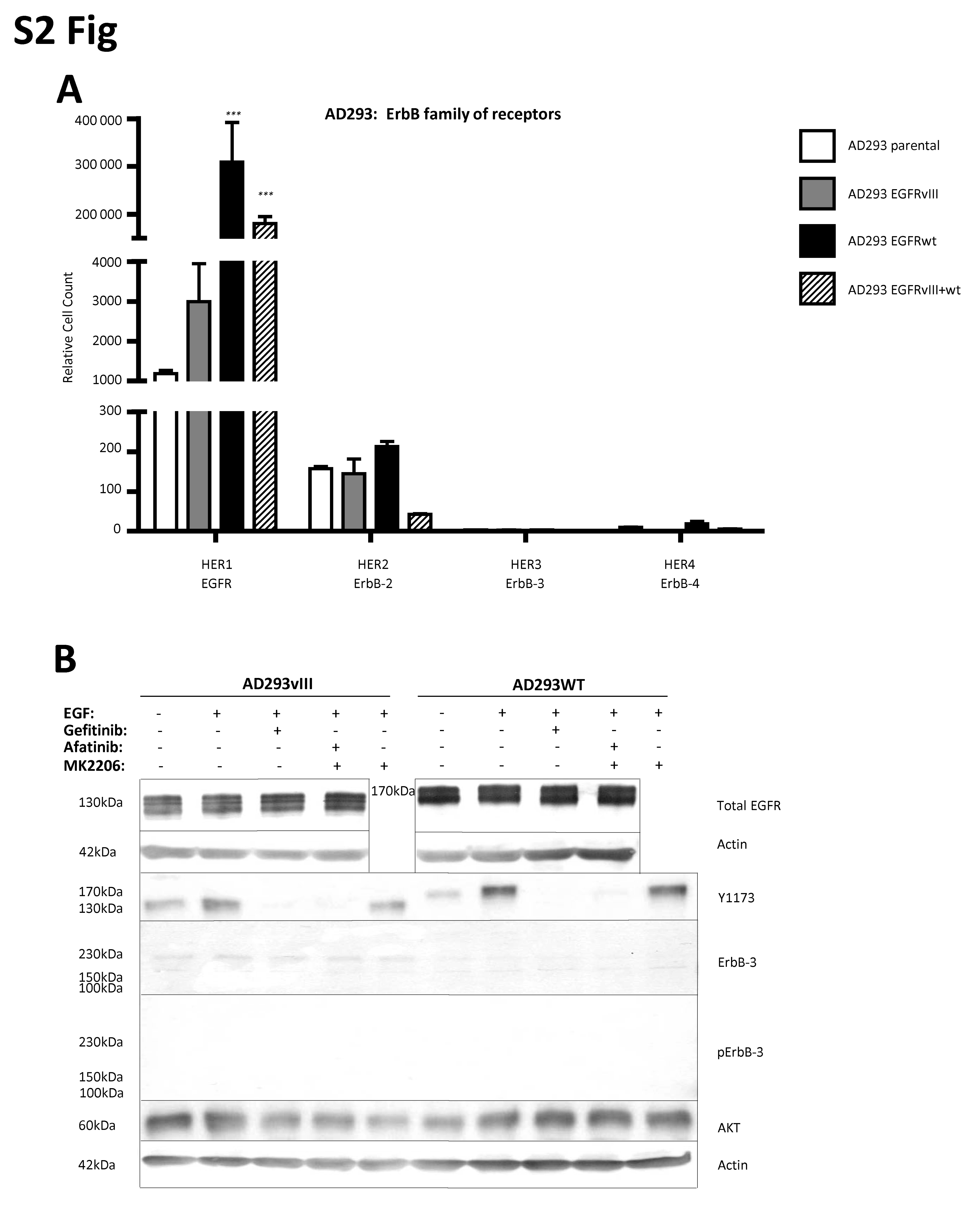

Supplement: S2 Fig — (A) ErbB family RTKs are expressed at a very low level in AD293 cell lines. Real-time PCR was used to evaluate relative gene copy number variation of total EGFR (wt, HER1), HER2, HER3 and HER4 against HPRT1 housekeeping gene in AD293 cell lines. (B) Western blot analysis confirmed insignificant level of ErbB-3 (HER3) as well as lack of active/phosphorylated form of HER3. AD293 cells expressing EGFRvIII or EGFRwt were serum starved for 24 hours and DMSO/EGF/EGF+(Afatinib 500 nM)/Gefitinib 5 μM) were added. After 1 hour cells were lysed and blotted for phospho-EGFR, total EGFR, HER3, phospho-HER3, AKT and Actin. Statistical significance calculated against values for any of 4 genes in AD293par, ***: P<0.001. (TIF) [file pone.0155230.s002.tif]

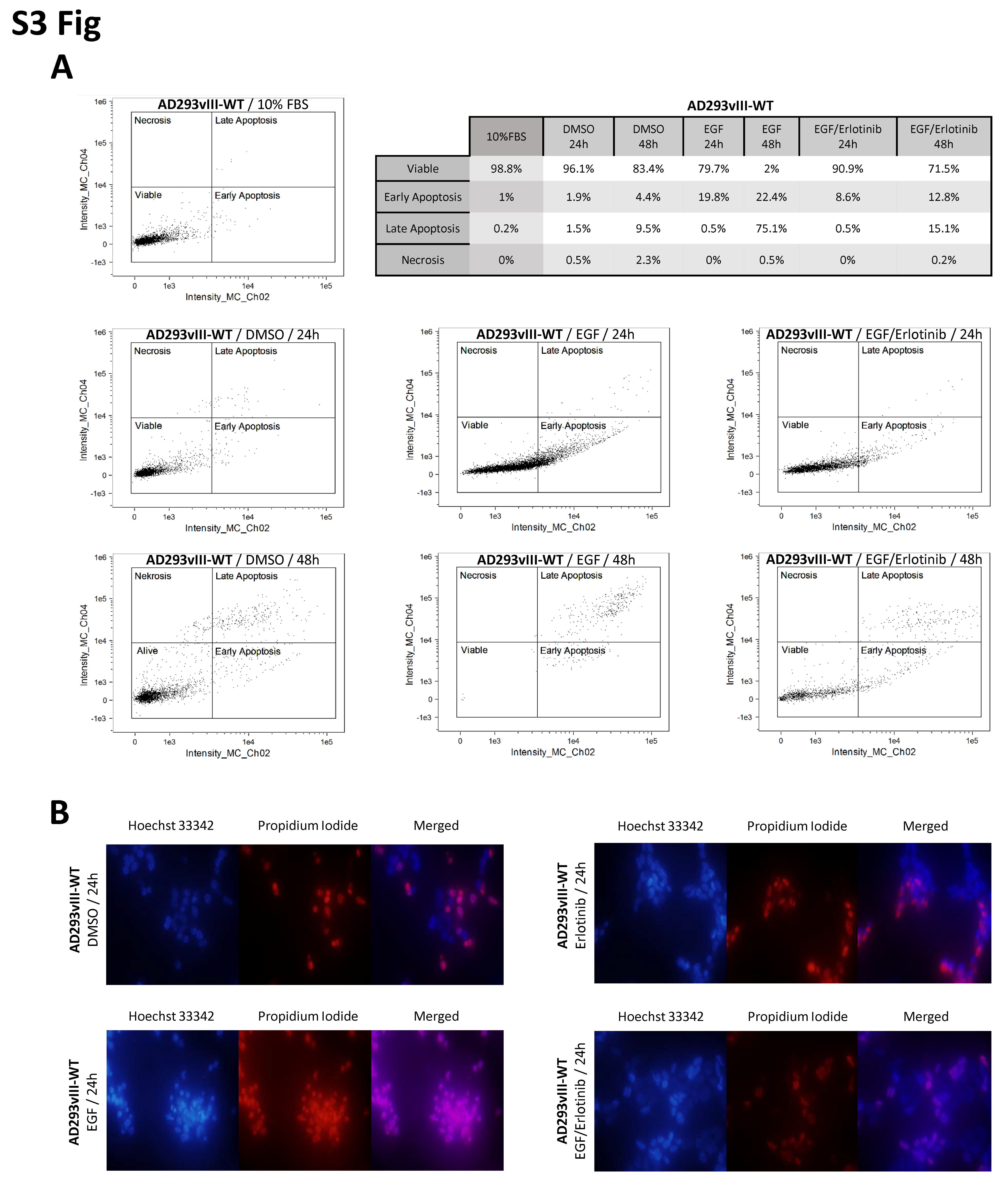

Supplement: S3 Fig — (A) Most of AD293 cells expressing both EGFRvIII and wild-type EGFR become apoptotic when treated with EGF. Medium was changed to serum free, DMSO/EGF/EGF+erlotinib was added, cells were harvested after 48 hours, stained with Annexin V FITC/propidium iodide and analyzed by flow cytometry. As a control, untreated cells grown in complete serum (10% FBS added) were analyzed. (B) Analysis of compacted state of chromatin in apoptotic cells confirmed the link between EGF activated EGFRwt and apoptosis. Medium was changed to serum free, supplemented with DMSO/EGF/EGF+erlotinib/erlotinib, after 24 hour incubation cells were stained with propidium iodide and Hoechst 33342, and analyzed under fluorescence microscopy. (TIF) [file pone.0155230.s003.tif]

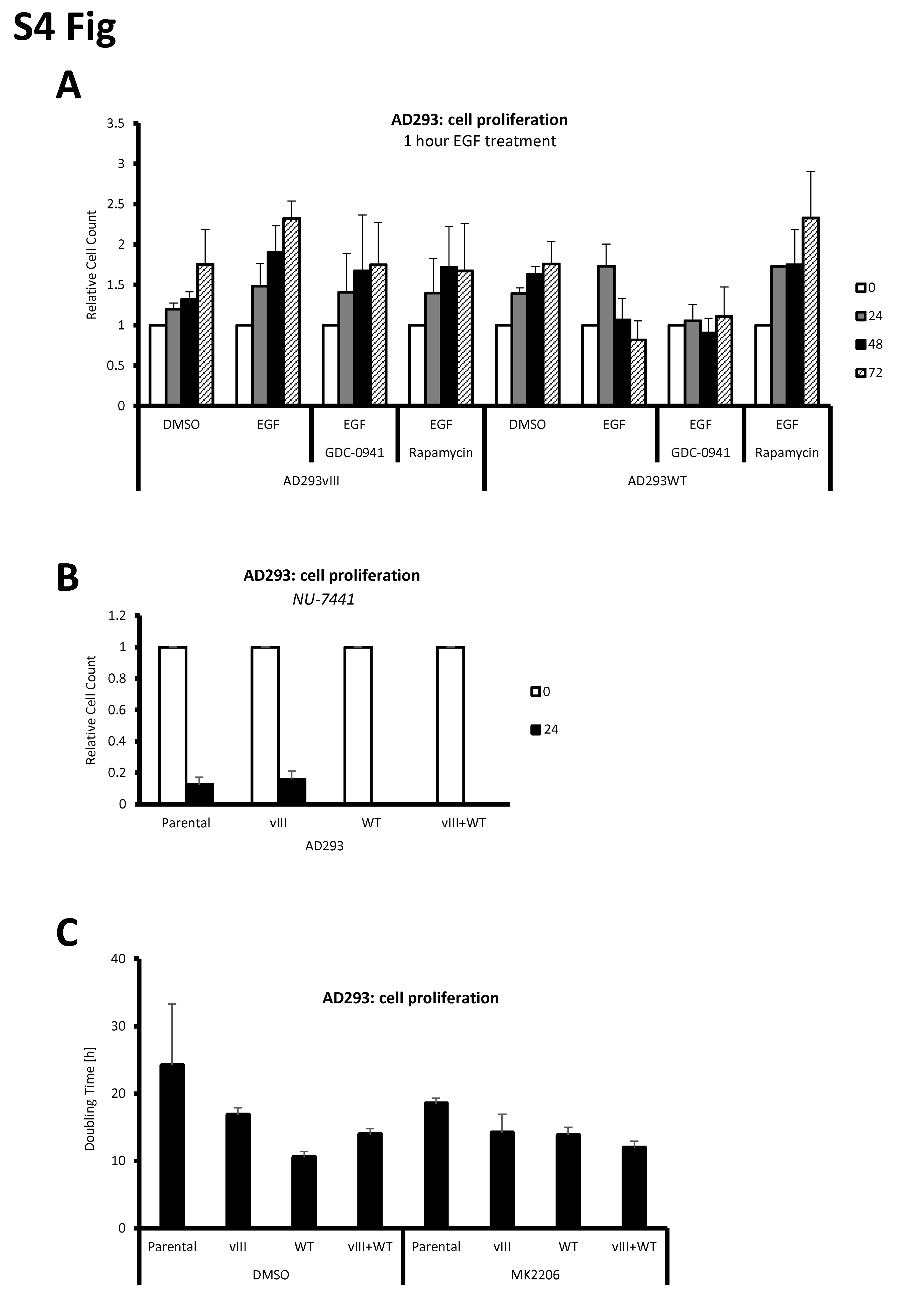

Supplement: S4 Fig — (A) Despite the peak of EGFRwt activity was observed after 1 hour treatment with EGF, this event was not sufficient to induce apoptosis/detachment of the cells. Medium was changed to serum free and supplemented with EGF (20 ng/ml) and DMSO or inhibitors (GDC-0941 1 μM; rapamycin 1 μM). After 1 hour medium was changed again and all of the abovementioned supplements were used, with the exception of EGF. Cells were photographed every 6 hours and counted (time points shown: 0 h, 24 h, 48 h and 72 h). Done in triplicates. (B) NU-7441, DNA-PK inhibitor, induces robust cell detachment within 24 hours. Medium was changed to serum free and supplemented with EGF (20 ng/ml) and inhibitor NU-7441 (1 μM). Cells were photographed at 0 h and 24 h time points, each time at least 3 wells were evaluated. (C) MK2206, AKT inhibitor, has no significant influence on cell proliferation in complete growth medium. Cells were seeded onto 6-well plate in complete medium and after 6 hours were moved to Nikon BioStation CT for live observation. (TIF) [file pone.0155230.s004.tif]
