# Supplementary material for: EGFR Activation Leads to Cell Death Independent of PI3K/AKT/mTOR in an AD293 Cell Line
Source: PLoS One. 2016 May 6;11(5):e0155230. doi: 10.1371/journal.pone.0155230 (PMC4859505; doi:10.1371/journal.pone.0155230)
Supplement: S1 File — (PDF) [file pone.0155230.s005.pdf]

Supplementary Materials and Methods

Reagents and antibodies

MK2206, afatinib, erlotinib, gefitinib, GDC-0941, rapamycin, NU-7441 were purchased from Selleck Chemicals (USA). EGF was used in concentration 20 ng/ml (Invitrogen, Carlsbad, USA). Primary antibodies: total EGFR antibody sc-03 (Santa Cruz Biotechnology, USA), phospho-EGFR Y1173, phospho-STAT5 Y694, phospho-AKT S473 and AKT #9272 (Cell Signaling Technology, USA), anti-Actin MAB1501 (Millipore, USA). Secondary antibodies: goat anti-rabbit (sc-2004) and goat anti-mouse (sc-2005) (SantaCruz).

DNA isolation

Total cellular DNA was isolated from fresh cell cultures at early passages using AllPrep DNA/RNA Mini Kit (Qiagen, Germany) according to the manufacturer’s protocol. DNA concentration was measured spectrophotometrically.

Multiplex Ligation-dependent Probe Amplification (MLPA)

The MLPA reactions were performed using the commercially available probe mixes (P105-D1 Glioma-2, MRC-Holland, Netherlands) and kits (SALSA MLPA EK1 reagent kit – FAM, MRC-Holland, Netherlands) according to the manufacturer’s protocol. In brief, 5 ml samples with 200 ng of genomic DNA were denatured at 98°C for 5 min and then cooled to 25°C. Next, 3 ml of hybridization mastermix (containing 1.5 ml of MLPA buffer and 1.5 ml of probemix per sample) was added to each sample and incubated at 98°C for 1 min and at 60°C for 16-18h. Next, without removing the tubes from the thermocycler (paused at 54°C), 32 ml of ligase mastermix (containing 25 ml water, 3 ml ligase buffer A, 3 ml ligase buffer B and 1 ml ligase per sample) was added to each sample and incubated at 54°C for 15 min and at 98°C for 5 min, then cooled to 20°C. Finally, 10 ml of polymerase mastermix (containing 7.5 ml water, 2 ml SALSA PCR primer mix and 0.5 ml polymerase per sample) was added to each sample and the cycling conditions were as follows: 35 cycles of denaturation at 95°C for 30 s, annealing at 60°C for 30 s and elongation at 72°C for 60 s followed by incubation at 72°C for 20 min. The products were cooled to 15°C and stored in a dark box at 4°C. Before capillary electrophoresis the injection plate was heated to 86°C for 3 min and cooled for 5 min at 4°C. The fragments were separated by capillary electrophoresis using ABI 3130 genetic analyzer (Applied BioSystems, capillaries length 50 cm). The comparative

analyzes were performed using the newest version of Coffalyzer.Net (MRC-Holland, Netherlands). For each gene, the resultant ratio was calculated and interpreted as a gain (more than 1.3) or loss (less than 0.7).

RNA isolation, reverse transcription and Real-time PCR

Total RNA was isolated from the cultures using NucleoSpin RNA Kit (Macherey-Nagel, Germany) according to the manufacturer’s protocol. RNA concentrations were measured spectrophotometrically (NanoPhotometer Pearl, Implen GmbH, Germany). Total RNA was reverse transcribed using a QuantiTect Reverse Transcription Kit (Qiagen, Germany) according to the manufacturer’s protocol. Quantitative Real-Time PCR reactions were performed using SYBR® Select Master Mix (Life Technologies) in StepOnePlus Real-Time PCR System (Applied Biosystems, USA) to determine expression of HER1, HER2, HER3, HER4 and HPRT1 genes (Primer sequences in S1 Table). HPRT1 gene was used as a reference gene. The cycling conditions were as follows: 2 min at 50°C (UDG activation), 10 min at 95°C (polymerase activation) followed by 40 cycles of: 15 s at 95°C (denaturation), 30 s at 60°C (annealing) and 30 s at 72°C (extension). The applied primer sequences are listed in S1 Table. Real-time PCR efficiency and the relative expression were calculated using LinReg software. Graph presents relative copy number variation between each gene.
