# Supplementary material for: EGFR Activation Leads to Cell Death Independent of PI3K/AKT/mTOR in an AD293 Cell Line
Source: PLoS One. 2016 May 6;11(5):e0155230. doi: 10.1371/journal.pone.0155230 (PMC4859505; doi:10.1371/journal.pone.0155230)
Supplement: S1 Table — (PDF) [file pone.0155230.s006.pdf]

S1 Table. Primer sequences.

Primer	Sequence
ErbB-2-F	TGGCCTGTGCCCACTATAAG
ErbB-2-R	AGGAGAGGTCAGGTTTCACAC
ErbB-3-F	GACCCAGGTCTACGATGGGAA
ErbB-3-R	GTGAGCTGAGTCAAGCGGAG
ErbB-4-F	GCCTCTGGAGAATTTACGCAT
ErbB-4-R	GGGTTCCGAACAATATCTTGCC
HPRT1-F	GACCAGTCAACAGGGGACAT
HPRT1-R	AACACTTCGTGGGGTCCTTTTC
